# CFH exerts anti-oxidant effects on retinal pigment epithelial cells independently from protecting against membrane attack complex

**DOI:** 10.1038/s41598-019-50420-9

**Published:** 2019-09-25

**Authors:** Céline Borras, Jérémie Canonica, Sylvie Jorieux, Toufik Abache, Mohamed El Sanharawi, Christophe Klein, Kimberley Delaunay, Laurent Jonet, Michèle Salvodelli, Marie-Christine Naud, Yvan Arsenijevic, Andrée Shalabi, Landry Souchaud, Francine Behar-Cohen, Virginie Dinet

**Affiliations:** 1 Centre de Recherche des Cordeliers, Université Pierre et Marie Curie - Paris6, UMRS 872, Paris, F-75006 France; 2 Université Paris Descartes, UMR S 872, Paris, F-75006 France; 3 INSERM, U1138, Paris, F-75006 France; 4 Université Paris Diderot, F-75007 Paris, France; 5 Department of Ophthalmology, University of Lausanne, Jules-Gonin Eye Hospital, Lausanne, Switzerland; 6 Laboratoire Français du Fractionnement (LFB), Paris, France; 7 Neurologie und Neurochirurgie, Uniklinik Frankfurt, Frankfurt, Germany

**Keywords:** Complement cascade, Visual system

## Abstract

Age Related Macular Degeneration (AMD) is the first cause of social blindness in people aged over 65 leading to atrophy of retinal pigment epithelial cells (RPE), photoreceptors and choroids, eventually associated with choroidal neovascularization. Accumulation of undigested cellular debris within RPE cells or under the RPE (Drusen), oxidative stress and inflammatory mediators contribute to the RPE cell death. The major risk to develop AMD is the Y402H polymorphism of complement factor H (CFH). CFH interacting with oxidized phospholipids on the RPE membrane modulates the functions of these cells, but the exact role of CFH in RPE cell death and survival remain poorly understood. The aim of this study was to analyze the potential protective mechanism of CFH on RPE cells submitted to oxidative stress. Upon exposure to oxidized lipids 4-HNE (4-hydroxy-2-nonenal) derived from photoreceptors, both the human RPE cell line ARPE-19 and RPE cells derived from human induced pluripotent stem cells were protected from death only in the presence of the full length human recombinant CFH in the culture medium. This protective effect was independent from the membrane attack complex (MAC) formation. CFH maintained RPE cells tight junctions’ structure and regulated the caspase dependent apoptosis process. These results demonstrated the CFH anti-oxidative stress functions independently of its capacity to inhibit MAC formation.

## Introduction

Age-related macular degeneration (AMD) is a complex multi-factorial degenerative disease that affects 50 million individuals worldwide and is the leading cause of vision loss in developed countries. Clinically, two advanced forms of AMD are recognized, the atrophic (aAMD) and the neovascular (nAMD) forms. Whilst anti-VEGF therapies are approved for nAMD, there is no validated treatment for aAMD^1^. Although photoreceptors die in the macula^2^, the initial pathogenesis of AMD, involves the degeneration of retinal pigment epithelial cells (RPE)^3^, which is preceded by deposits between the RPE and the Bruch membrane (BrM), recognized as drusen, a whole mark of AMD. The RPE forms a monolayer of support cells essential for photoreceptor functions, ensuring retinoid cycle, phagocytosis of photoreceptor outer segments and for maintaining the blood-retina barrier, which is disrupted during nAMD^4^. Because of the close interaction between RPE and photoreceptors in both nutritional and metabolic aspects^5^, RPE dysfunction is associated with photoreceptor degeneration.

RPE cells are permanently submitted to oxidative stress as daily amount of oxidized phospholipids shed by photoreceptors are engulfed by RPE during phototransduction^6,7^. Exacerbated oxidative stress in RPE contributes to AMD^8^ eliciting decomposition of polyunsaturated fatty acid and the formation of 4-hydroxy-2-nonenal (4-HNE), a highly reactive but relatively stable end-product of lipid peroxidation which directly contributes to oxidative cell damage^9,10^. Substantial evidence indicates that 4-HNE production and their deleterious effects are associated with AMD^9–12^. The polymorphism Y402H of the complement factor H (CFH) has been strongly associated with a risk of developing AMD^13–16^. CFH is the major inhibitor of the complement alternative pathway. It interferes with the formation and activity of the C3 convertase (C3bBb), decreases the C5b9 membrane-attack complex (MAC) formation and the two anaphylotoxins C3a and C5a. Three major regions of the 20 existing complement control protein (CCP) modules of CFH are essential for its activity and its surface binding. The CCPs1-4 modules play a crucial role in the regulation of the anti-C3 convertase activity of CFH while CCPs6-8 and CCPs18-20 carry two CFH binding sites to heparin/glycosaminoglycans (GAGs) on host cells, protecting them against complement activation^17–19^. The Y402H CFH variant reduced CFH ability to neutralize oxidized lipids, enhancing their toxic and inflammatory effects^20,21^ and demonstrating a relationship between CFH and oxidative stress.

RPE cells are a major source of complement activator and inhibitor factors at the retina-choroid interface and in subretinal space^22^. Photo-oxidative damaged RPE cells directly activate different complement components^23,24^. The mechanisms that link the complement system and oxidative damage of RPE in early stages of AMD are insufficiently understood. A critical role of C3 has been demonstrated in the formation of sub-RPE deposits^25,26^ and C3a, the cleavage product of C3 induced by the activation of alternative pathway stimulates deposition of collagens IV and VI underneath the RPE and impairs the extracellular matrix turnover^27^. The binding of C3a on the RPE membrane cells is also associated with oxidative stress and calcium mobilization, reticulum stress, and VEGF secretion^28,29^. Sublytic levels of MAC alter the RPE barrier integrity, and induce secretion of VEGF and of pro-inflammatory cytokines^30,31^. Additionally, Ramos and collaborators have demonstrated an association between C3a production and proteolytic activity of the proteasome in a mouse model of age-related RPE atrophy^32^. All together, these data suggest a link between oxidative stress, activation of AP and degeneration of RPE.

To better understand the role of CFH in degenerative processes underlying RPE death, we analyzed the effects of CFH and the contribution of its CCP domains on the RPE death induced by oxidative stress (4-HNE). We found that the three functional domains of CFH, CCPs1-4 anti-C3 convertase and its two binding domains CCPs6-8 and CCPs19-20 are mandatory to protect RPE cells from oxidative stress-induced cell death. CFH maintained the tight junction integrity. The protecting effect of CFH was independent from the inhibition of MAC formation and was associated with regulation of caspase-dependent apoptosis pathway.

## Results

### Only full length CFH protects RPE from oxidative stress-induced cell death

**Figure 1:**
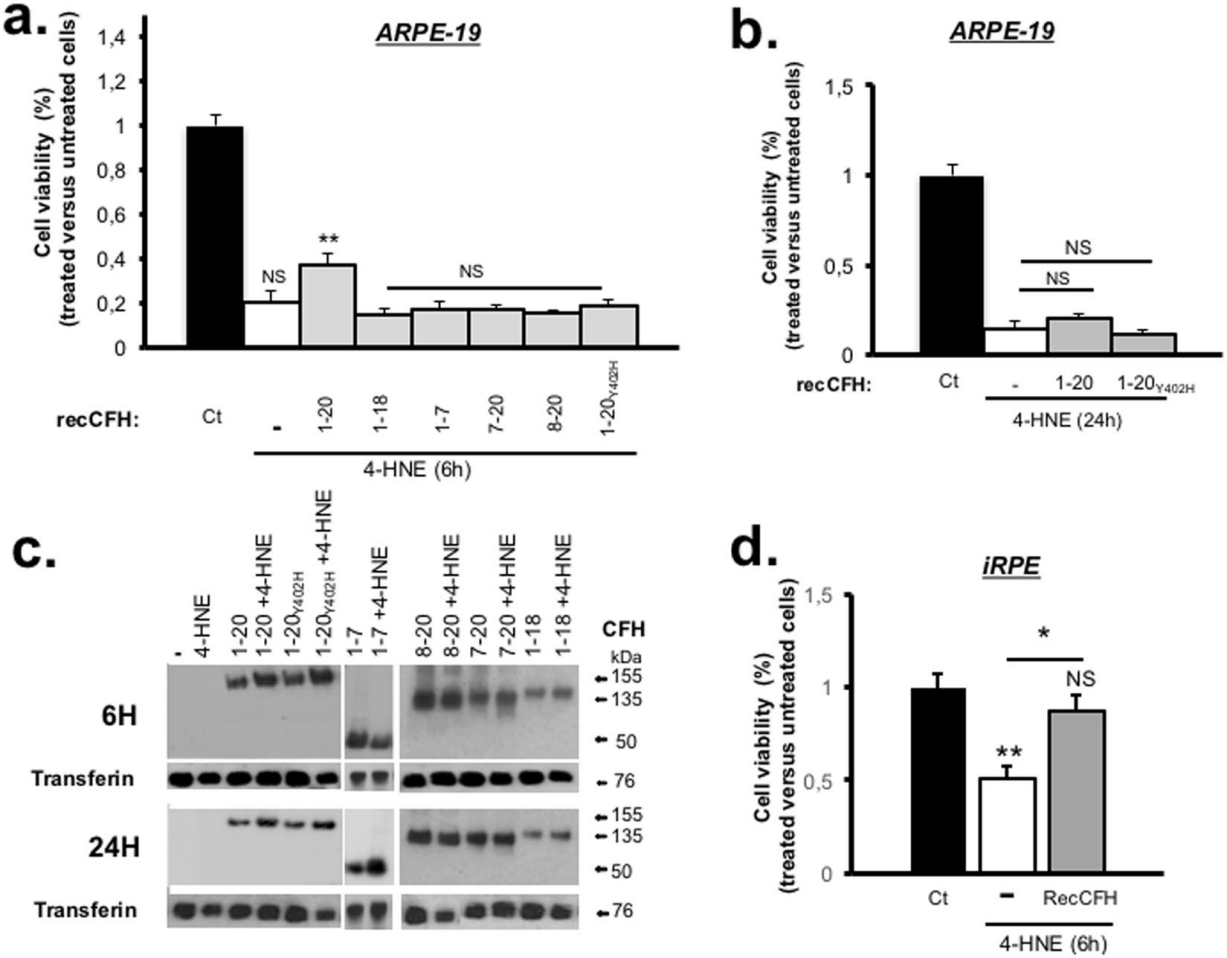
CFH full length promotes RPE cell survival under oxidative stress treatment. (**a**,**b**) Exposure of 4-HNE (30 μM) induced at least 70% ARPE-19 cells death after 6 h of culture. Analysis of recCFH (300 nM) effect on survival ARPE-19 cells 6 h **(a)** or 24 h **(b)** after 4-HNE (30 μM) treatment. The protective effect of recCFH full length observed at 6 h was abolished 24 h after 4-HNE treatment. No protective effect was observed with recCFH_Y402H_ or recCFH fragments 6 h after treatment. **(c)** Western blotting showed the disponibility of recCFH and its fragments in the ARPE-19 cells culture medium 6 h or 24 h after 4-HNE and recCFH co-treatment. The groupings of gels cropped were from different parts of the same gel, or from different gels separated by white line. **(d)** Analysis of hiPSC-derived RPE cells (iRPE) viability 6 h after 4-HNE (30 μM) treatment. RecCFH (300 nM) protected iRPE cells from 4-HNE cells death. All data were presented as mean ± s.e.m. Statistical significance was assessed using Mann-Whitney test. *P < 0.05; **P < 0.01; NS = no significant.

The effects of recCFH (300 nM), added at the time of 4-HNE (30 µM) exposure, was evaluated after 6 and 24 hours. The 4-HNE dose of 30 µM was chosen as it induced more than 50% of an ARPE-19 cells death (Supplemental Fig. 1a). A hundred times dose was chosen for recCFH (fragments or full length). Concentration of 4-HNE was chosen to reflect *in vivo* exposure as it was shown to accumulate in membranes at concentrations ranging from 10 µM to 5 mM in response to oxidative stimuli^33^. We first showed that recCFH or recCFH fragments had no effect on ARPE-19 cells viability in control conditions (Supplemental Fig. 1b,c). Following 6 hours of culture, viable cells were counted using the trypan blue-excluding cell assay. Exposure of ARPE-19 to 4-HNE (30 µM) induced at least 70% ARPE-19 cells death at 6 hours compared to untreated cells (Fig. 1a). Addition of recCFH (300 nM) in the culture medium protected ARPE-19 cells from death by 56% (P < 0.01) as compared to cells treated only with 4-HNE (Fig. 1a). This protection was abolished after 24 hours of culture and was associated with a decrease in the amount of recCFH in the culture medium (Fig. 1b,c). To identify the CCPs domains of recCFH, carrying the antioxidant activity, we tested several recCFH fragments. Because CCPs1-4 domains are essential for the anti-C3 convertase activity of CFH and both CCPs6-7 and CCPs19-20 are important for CFH membrane binding, we decided to test recCFH1-18 (without binding site CCPs19-20), recCFH 8–20 (with only the CCPs19-20 binding site), recCFH 1–7 (contains both anti-C3 convertase CCPs1-4 domains associated to binding site CCP7) and recCFH 7–20 (contains both binding sites without anti-C3 convertase domains). After 6 hours, none of the recCFH fragments significantly protected ARPE-19 cells from death induced by 4-HNE (Fig. 1a), despite their presence in the culture medium (Fig. 1c). Thus, only the full length recCFH was effective to protect RPE cells from 4-HNE-induced cell death. Contrariwise, the co-treatment of 4-HNE and recCFH_Y402H_, carrying the Y402H polymorphism, did not protect ARPE-19 cells from death, despite its presence in the culture medium (Fig. 1a–c). The protective effect of full length recCFH was investigated in hiPSC-derived RPE (iRPE) cells. iRPE cells grown in monolayers of polygonal pigmented cells (Udry *et al*., submitted), express most of RPE biomarkers (e.g. RPE65, RLBP1 and BEST) and show phagocytic ability. RecCFH also protected iRPE cells from 4-HNE toxicity (Fig. 1d).

**Figure 2:**
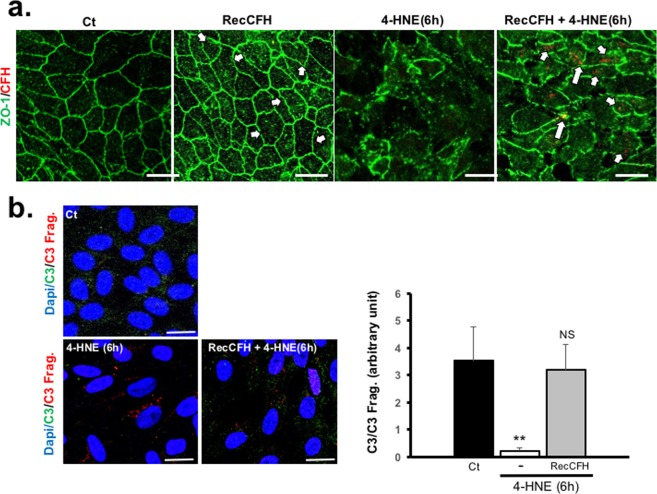
CFH is internalized by ARPE-19 cells upon 4-HNE treatment. (**a)** CFH and ZO-1 immunostaining on ARPE-19 cells 6 h after 4-HNE (30 μM) with or without recCFH (300 nM) co-treatment. **(b)** C3 and C3 fragments (C3Frag.) co-immunostaining 6 h after exposure to 4-HNE (30 μM) or to 4-HNE (30 μM) and recCFH (300 nM) on ARPE-19 cells. Semi-quantification of C3 and C3 Frag. immunostaining showed less C3 cleavage in ARPE-19 cells co-treated with recCFH compared to 4-HNE treatment. All data were presented as mean ± s.e.m. Statistical significance was assessed using Mann-Whitney test. **P < 0.01; Scale bars: 50 μm.

**Figure 3:**
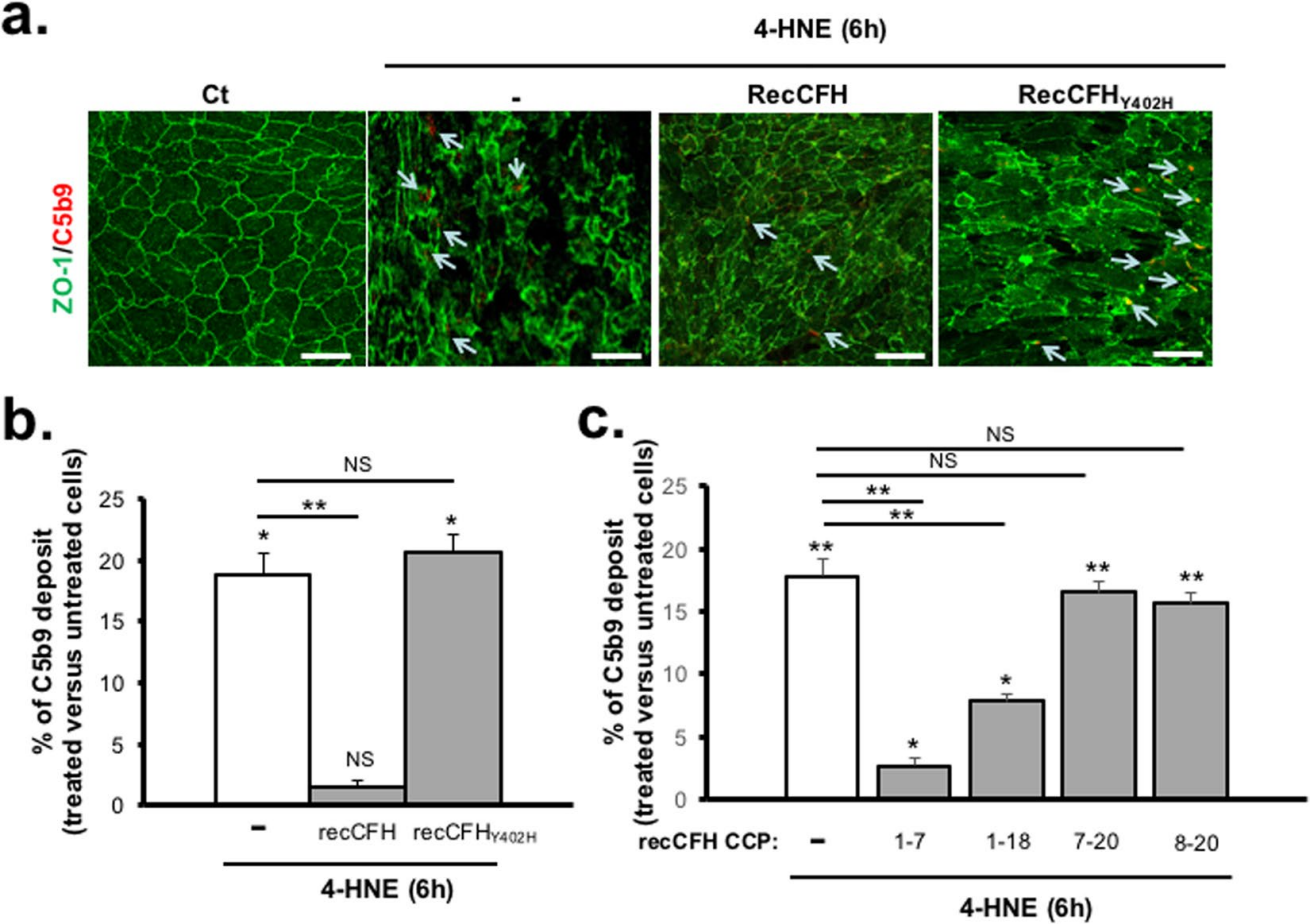
CFH protects ARPE-19 cells from oxidative stress independently of reduced MAC deposit. (**a)** C5b9 immunostaining on ARPE-19 cells 6 h after 4-HNE (30 μM) with or without recCFH or recCFH_Y402H_ (300 nM) co-treatment. **(b)** As compared to 4-HNE treatment, semi-quantification of C5b9 immunostaining showed less MAC formation in ARPE-19 cells co-treated with recCFH full length, in contrast to its mutated form recCFH_Y402H_. **(c)** Semi-quantification of C5b9 immunostaining revealed less MAC deposit with recCFH 1–7 or 1–18 but not with recCFH 7–20 or 8–20 fragments as compared to 4-HNE treatment only. All data were presented as mean ± s.e.m. Statistical significance was assessed using Mann-Whitney test. *P < 0.05; **P < 0.01; NS = no significant. Scale bars: 50 μm.

RecCFH added in the culture medium was found, not only, on the ARPE-19 cell membrane upon 4-HNE treatment, but also, in the cytosolic compartment (Fig. 2a). Once in contact with ARPE-19 cells exposed to 4-HNE treatment, recCFH protected C3 from cleavage to its C3 fragments (C3 Frag.), resulting in a higher C3/C3 Frag. ratio as compared to 4-HNE treated cells (Fig. 2b). Exposure to 4-HNE increased by 19% the deposit of MAC on ARPE-19 cell membranes, identified by C5b9 immunodectection (Fig. 3a,b). Treatment with full length recCFH significantly prevented MAC deposition_,_ but treatment with its polymorphism form recCFH_Y402H_ did not (Fig. 3a,b). Although recCFH 1–7 and 1–18 did not protect from death cells exposed to 4-HNE, they significantly decreased MAC deposit on ARPE19 cells by respectively 86% and 56% (Fig. 3c), suggesting that CFH protection resulted from mechanisms unrelated to MAC deposit (Figs 1a and 3c).

### CFH protects RPE tight junctions from oxidative stress-induced disruption

**Figure 4:**
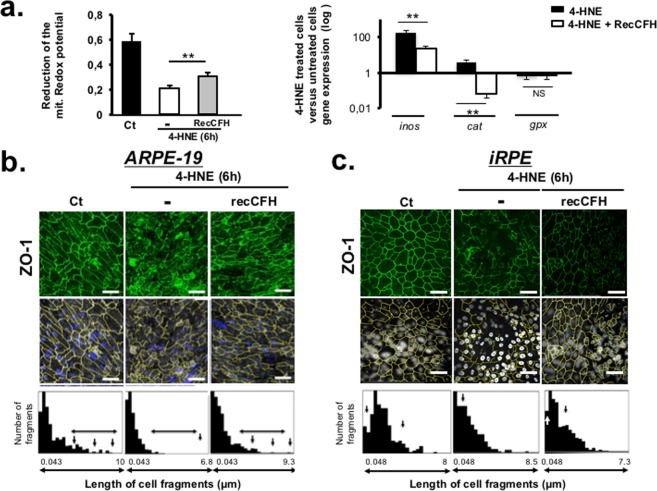
CFH protects RPE cells tight junctions from oxidative stress. (**a)** ARPE‐19 cultures were treated with 4-HNE (30 μM) in the presence or not of recCFH (300 nM) and the mitochondrial redox potential was analysed by the MTT colorimetric method 6 h after. *Inos*, *Catalase (cat*) and *Gpx* mRNA expression were investigated by reverse transcription quantitative polymerase chain reaction (RT-qPCR) experiments 6 h after 4-HNE (30 μM) or 4-HNE (30 μM) and recCFH (300 nM) ARPE-19 cells co-treatment. All data were presented as mean ± s.e.m. Statistical significance was assessed using Mann-Whitney test. *P < 0.05; **P < 0.01; NS = no significant. Scale bars: 50 μm. **(b**,**c**) ZO-1 immunostaining was altered in ARPE-19 **(b)** and iRPE **(c)** cells 6 h after 4-HNE (30 μM) treatment. Exposure to recCFH (300 nM) preserved ZO-1 immunostaining in both ARPE-19 and iRPE 4-HNE treated cells. Quantification of ZO-1 immunostaining length fragment showed longer immunostained fragments only with recCFH as compared to 4-HNE treatment for both ARPE-19 and iRPE cells. Scale bars: 50 μm.

We first used an MTT (3 (4,5‐dimethylthiazol‐,yl) 2,5 diphenyl tetrazolium bromide) colorimetric assay to investigate the effect of recCFH on the mitochondrial redox potential of ARPE‐19 cultures. Co-treatment with 4-HNE and recCFH reduced the decrease previously observed with 4-HNE treatment in the mitochondrial redox potential of these cells (Fig. 4a). Upon 4-HNE treatment, a lower variation of anti-oxidative gene expression (*Catalase*) was seen, while a significant increase of *inos* oxidative gene expression in ARPE-19 cells could be observed as compared to untreated cells (Fig. 4a). RecCFH prevented the 4-HNE-induced regulation of pro-and anti-oxidative genes (Fig. 4a). One of RPE functions is to maintain the outer blood-retina barrier by expressing tight and adherence junction proteins, such as ZO-1. 4-HNE treatment altered ZO-1 immunostaining at ARPE-19 (Fig. 4b) and iRPE (Fig. 4c) cell membranes. RecCFH protected RPE cells junction integrity (Fig. 4b,c), as quantified by count the number of ZO-1-immunolabeled fragments according to their length (Fig. 4b,c). The protective effect of recCFH from oxidative stress on the ARPE-19 or iRPE cells structure was confirmed by immunofluorescent experiments using Phalloidin with or without ZO-1 co-labeling (Supplemental Fig. 2).

### CFH preserves mitochondria and nucleus structure of RPE submitted to oxidative stress

**Figure 5:**
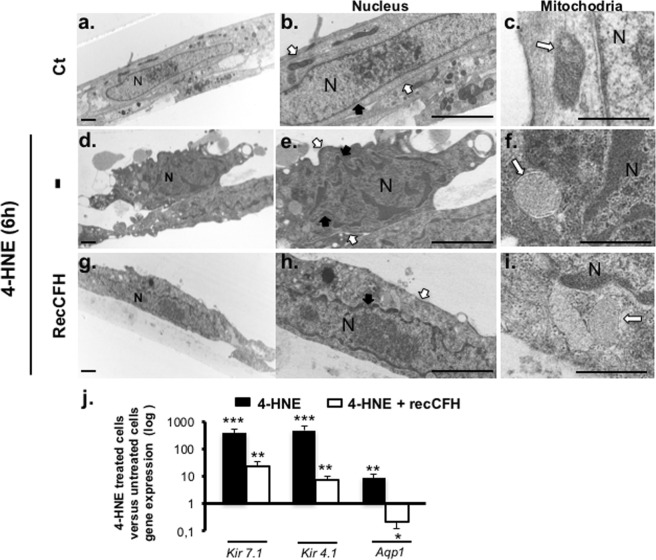
CFH protects RPE cells morphology and ultrastructure against oxidative stress. **(a–c)** Electron micrographs of ARPE-19 cells 6 h after **(d–f)** 4-HNE (30 μM), **(g–i)** 4HNE (30 μM)/recCFH (300 nM) treatments were analyzed. Arrows indicated the nucleus membrane form (black arrows) or the mitochondrial morphology (white arrows). Mitochondrial morphology, nucleus form and volume were protected by recCFH in ARPE-19 cells co-treated with 4-HNE. Scale bars: 2 μm. **(j)** *Kir7.1*, *Kir4.1* and *Aqp1* mRNA levels were determined by reverse transcription quantitative polymerase chain reaction (RT-qPCR) 6 h after treatment with 4-HNE (30 μM) or with 4-HNE (30 μM) and recCFH (300 nM). RecCFH regulated osmotic flow in 4-HNE ARPE-19 cells treated by reducing the expression of *Kir7.1*, *Kir4.1* and *Aqp1* mRNA levels. All data were presented as mean ± s.e.m. Statistical significance was assessed using Mann-Whitney test. **P < 0.01; ***P < 0.005.

Using electron microscopy, exposure of ARP-19 to 4-HNE induced morphological features of apoptotic cells, including chromatic margination, nuclear condensation and cell fragmentation in apoptotic membrane-bound bodies (Fig. 5a,b,d,e). Treatment with recCFH preserved the normal cell nucleus morphology (Fig. 5g,h). Dynamic remodeling of mitochondrial morphology is also an important indicator of healthy cells. In 4-HNE-treated ARPE-19 cells, mitochondria showed fractured tubular cristae with a round form compared to untreated ARPE-19 cells, which had many of continuous tubular cristae and an egg-shaped form (Fig. 5c,f). Co-treatment with 4-HNE/recCFH preserved the framework of mitochondria (Fig. 5c,i). All together, these data demonstrated a protective effect of CFH on oxidative stress-induced cellular organites. Because the cellular volume of ARPE-19 cells co-treated with 4-HNE and recCFH was reduced compared to 4-HNE treatment cells (Fig. 5e,h), the expression profile of genes implicated in osmotic flow was investigated. 4-HNE treatment up-regulated *Kir7.1* and *Kir4.1*potassium channel and aquaporin 1 (*Aqp1*) gene expression, while recCFH reduced significantly these gene expression (Fig. 5j).

### CFH protects RPE from caspase-dependent apoptosis

**Figure 6:**
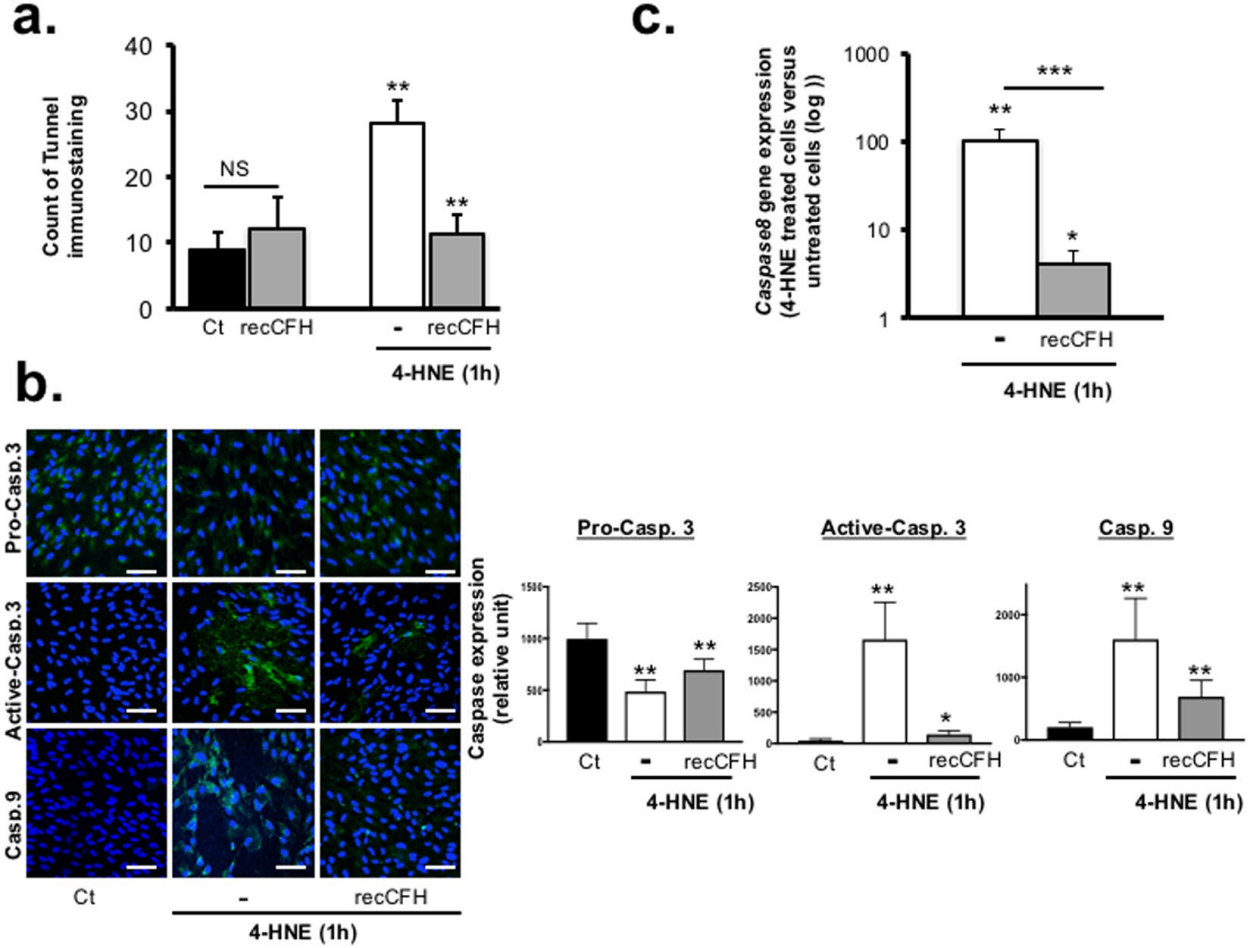
CFH regulates caspase dependent apoptosis. (**a)** TUNEL staining was performed and quantified in ARPE-19 cells 1 h after 4-HNE (30 μM) or after 4-HNE (30 μM) and recCFH (300 nM) treatment. RecCFH protected ARPE-19 cells from apoptosis **(b)** Immunostaining of pro-caspase3, active caspase 3 and caspase 9 was performed 1 h after ARPE-19 4-HNE or 4-HNE/recCFH treatment (30 μM). Semi-quantification of caspase-immostaining showed an increase of pro-caspase 3 cleavage by caspase 9 in ARPE-19 upon 4-HNE treatment as compared to a co-treatment with recCFH. **(c)** Reverse transcription quantitative polymerase chain reaction (RT-qPCR) analysis showed a decrease of *caspase 8* mRNA expression in ARPE-19 cells 1 h after treatment in presence of recCFH (300 nM) as compared to 4-HNE (30 μM) treatment alone. All data are presented as mean ± s.e.m. Statistical significance was assessed using Mann-Whitney test. *P < 0.05; **P < 0.01; ***P < 0.005, NS = no significant. Scale bars: 50 μm.

Mechanisms of CFH on 4-HNE-induced cell death were studied. RecCFH reduced the number of TUNEL positive cells by 60% (*vs*. 4-HNE treatment, p < 0.001) (Fig. 6a). To explore whether apoptosis was caspase dependent, we measured the levels of capsase3 activation using immunohistochemistry. In untreated ARPE-19 cells, pro-caspase3 immunolabelling was observed in contrast to active caspase3 and caspase9 (Fig. 6b). Exposure to 4-HNE increased the immunolabelling signal of caspase9 and active-caspase3 (Fig. 6b). Co-treatment with recCFH reduced caspase9 and active caspase3, as shown by semi-quantified immunostaining (Fig. 6b). Pro-caspase3 is activated in the apoptotic cell death both by extrinsic (death ligand cascade involving caspase 8) and intrinsic (mitochondrial cascade implicating caspase9) pathways. In this study, expression of *caspase 8* mRNA was also reduced 25 times (p < 0.05) on RT-qPCR compared to 4-HNE ARPE-19 cells treatment (Fig. 6c). These data show that CFH regulated both extrinsic and intrinsic apoptosis pathways by modulating caspases expression.

### CFH protects RPE from necrosis

**Figure 7:**
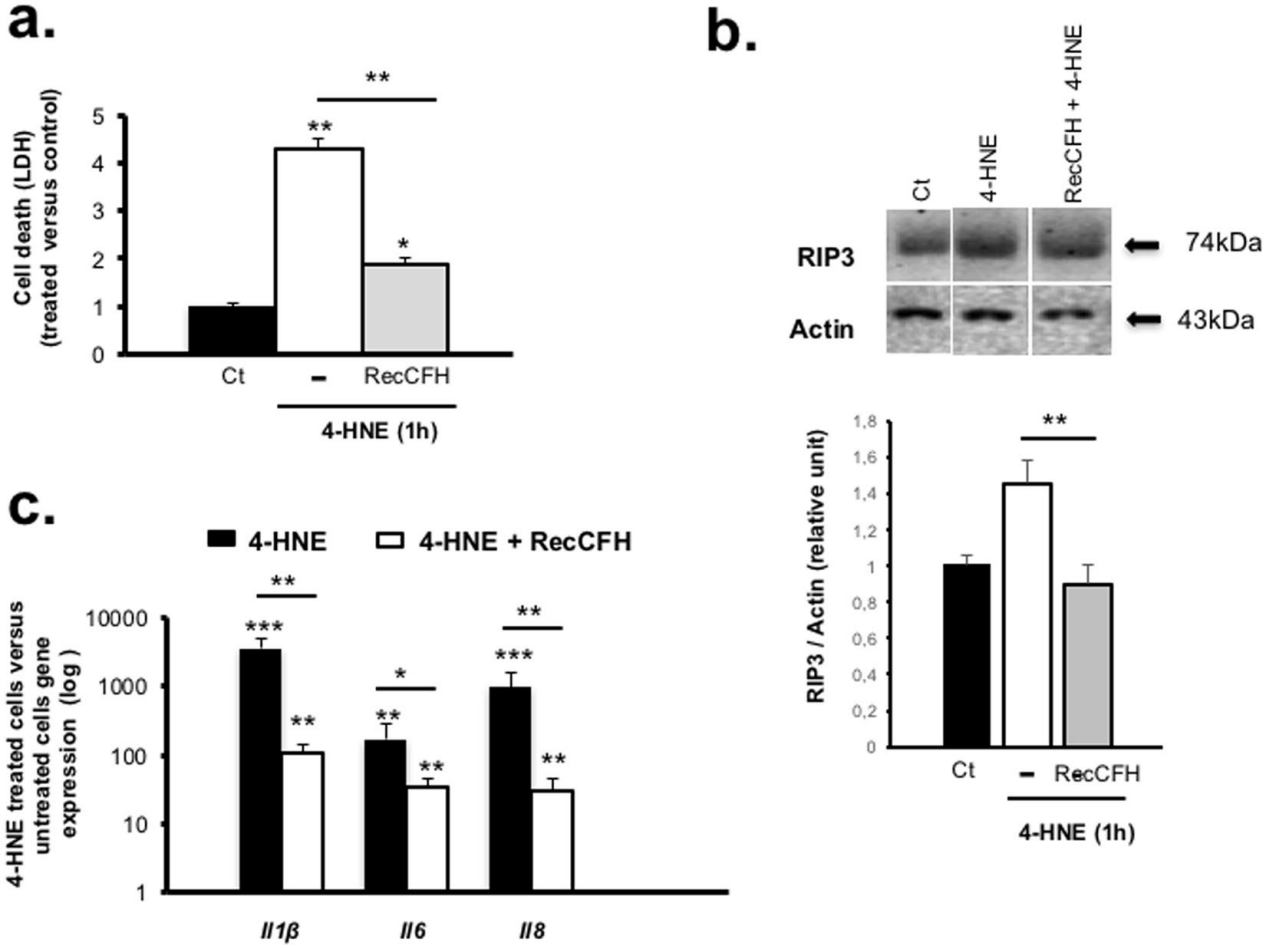
CFH reduces necrotic process in oxidative stress conditions. (**a)** Lactate dehydrogenase (LDH) release was measured 1 h after 4-HNE (30 μM) treatment. LDH release was lower in culture medium contained recCFH (300 nM). **(b)** Necrotic RIP3 kinase protein level, detected by Western blotting, was reduced in ARPE-19 cells 1 h after 4-HNE (30 μM) treatment contained recCFH (300 nM). The groupings of gels cropped were from different parts of the same gel, or from different gels separated by white line **(c)** Reverse transcription quantitative polymerase chain reaction (RT-qPCR) analysis showed a decrease of *Il1β*, *Il6* and *Il8* mRNA expression in ARPE-19 cells 1 h after treatment with recCFH (300 nM) compared to 4-HNE alone (30 μM). All data are presented as mean ± s.e.m. Statistical significance was assessed using Mann-Whitney test. *P < 0.05; **P < 0.01; ***P < 0.005.

Necrosis is a type of cell death morphologically characterized by swelling, rupture of intracellular organelles, and cell membrane permeabilization, measured by the release of lactate dehydrogenase (LDH). Compared to untreated ARPE-19 cells, exposure to 4-HNE showed a 430% increase of LDH levels in culture medium (Fig. 7a). Treatment with recCFH reduced the LDH increased by 56% (P < 0.01) (Fig. 7a). On western-blot, the levels of receptor-interacting protein kinase 3 (RIP3), identified as a crucial regulator of death receptor-induced necrosis, was decreased by 36% (P < 0.01) in ARPE-19 cells in presence of recCFH compared to 4-HNE only (Fig. 7b). In addition, necrotic cells induce pro-inflammatory cytokines. Quantitative mRNA expression measurements revealed a major increase of several interleukins (*Il1β, Il6* and *Il8*) in 4-HNE-exposed cells compared to untreated cells (Fig. 7c). RecCFH treatment significantly reduced the expression of these inflammatory mediators (Fig. 7c).

## Discussion

Oxidative stress is a recognized pathogenic factor in the complex and multi-factorial occurrence of AMD. CFH was previously shown to protect RPE from hydrogen peroxide^34^, but the exact mechanisms of CFH on oxidative stress-induced damages in RPE has remained imperfectly understood. In this study, we showed that only the full length CFH protected RPE cells from death, contrariwise, this effect was abolished by the polymorphism CFH_Y402H_, demonstrating the importance of the CFH-CCP7 domain binding site. The binding CCP7 seems mandatory to mediate protection against 4-HNE-induced cell death. Factor H, the main alternative complement pathway (AP) regulatory protein that circulates in the plasma, controls AP activation and MAC formation on the surface of host cells through its interaction with GAGs, anionic molecules and complement C3 fragment displayed on the cell membrane^35^. In this study, we showed that full length recCFH reduced C3 cleavage and C5b9 deposit on ARP19 cell surface upon 4-HNE treatment, demonstrating a CFH functional activity on AP activation and MAC formation. The CFH domains (CCPs1-4), that has regulatory effect on the AP activation but do not bind on cell membrane, did not protect RPE from oxidative stress death. On the other hand, reduction of C5b9 deposit did not seem to be the major mechanism of cell death inhibition as there was no correlation between MAC and cell death inhibition using the different CFH fragments. Indeed, to be active on cell death, CFH needed to have the AP-regulatory domains responsible for the MAC formation and its two binding sites (with an intact CFH-CCP7 domain). Consistent with our data, it has been shown that protection against cell death is not achieved with CFH anti-C3 convertase domains^36^, and that it requires a cooperative bivalent binding of CFH at the cell membrane surface^37^. The CCP7 domain seems to play a major role as the recCFH_Y402H_, carrying the Y402H polymorphism, did not show any protecting effect, which could be one of the mechanisms of susceptibility to AMD in the population carrying this polymorphic variant.

The identification of CFH ligands at the surface of apoptotic cells remains unclear. Lipids are unlikely to be ligands for CFH on apoptotic cells^36^ but the calcium-dependent phospholipids-binding protein Annexin-II, involved in communication between cell membranes and the cytoplasm and in membrane trafficking and remodeling^38^ could be a CFH binding partner on apoptotic cells^36,39^. Interestingly, using different constructs of CFH, Leffler and collaborators show that fragments comprising CCPs6-8 and CCPs19-20 bind on apoptotic cells surface with higher affinity than one binding site^36^, consistent with a stronger survival effect of full length CFH.

Despite complement activation by down regulation of membrane bound complement regulatory proteins expression, apoptotic cells do not undergo lysis process^40^, consistent with a MAC independent death of ARPE-19 cells upon 4-HNE treatment. This complement death protection comes from activity of fluid phase complement inhibitors C4b-binding protein and CFH which limit C9 deposition on apoptotic cells membrane^40^. On other hand, **t**he binding of CFH on apoptotic cell surface triggers the activation of classic complement pathway C1 complex which ensures C3 cleavage to C3b responsible for the opsonization and removal of apoptotic cells debris^41^. In late apoptotic cells phase, CFH is internalized and acts as a cofactor for cathepsin L in the cleavage of C3 to opsonin iC3b which thereby facilitates phagocytosis of dying cells^42^. In this current study, we showed that ARPE-19 cells were protected from oxidative stress death independently of MAC formation inhibition but by specific functions of CFH. Indeed, CFH maintained RPE tight junctions and decreased caspase activation pathway just after exposure to oxidative stress. It has been demonstrated that activation of Caspase3 and C-Jun-N-terminal kinase (JNK) are observed in many 4-HNE induced apoptosis cell lines^43^. In this study, we showed that CFH down regulated, two pro-activators of Caspase3, Caspase8 and 9 expression previously inducing by exposure to 4-HNE. CFH regulated apoptotic cells death by modulating both extrinsic (caspase8) and intrinsic (caspase 9) apoptotic process. All together these data indicate that exposed to oxidative stress, CFH protected RPE tight junctions and reduced caspase activation pathway in these dying cells, but when damages were so high CFH was internalized and facilitated the removal of apoptotic cells by producing iC3b^42^. CFH binding domains were shown to have stronger attachment to necrotic cells as compared to apoptotic cells^36^, suggesting also a protective effect of CFH against necrosis. In our experiments, full length CFH also protected cells from necrosis as shown by the down regulation of RIP3 expression and LDH measurement.

It is recently shown that CFH, actively internalized by apoptotic RPE cells, forms complexes with nucleosomes which facilitates their phagocytosis by monocytes, ensuring an efficient removal of dying cells^42^. The binding between CFH with nucleosomes also modulates phagocytes cytokines towards an anti-inflammatory profile^42^. In our experiments, CFH also reduced the expression of pro-inflammatory cytokines by RPE cells, in agreement with a demonstrated reduction of Il-8 by CFH on malondialdehyde-acetaldehyde-induced RPE cell death^21^. However, CFH has no effect on Il-8 expression induced by phorbol myristate acetate oxidative stress^21^, suggesting that the CFH inflammatory regulation is dependent on the nature of oxidative stress.

In conclusion, this study showed that only full length CFH protected both human ARPE-19 cell line and iRPE from oxidative stress-induced cell death created by exposure to 4-HNE. Both necrosis and caspase-dependent apoptosis were reduced by CFH. Exposure to 4-HNE increased MAC deposit on RPE cells while full length CFH as well as CCPs1-7 and CCPs1-18 decreased MAC, but only full length CFH protected from oxidative stress-induced cell death, suggesting an effect independent from MAC formation. The Y402H polymorphism form of CFH, that is associated with the risk of AMD, lost the protective effect. Taken together, these results suggest that CFH per se exerts antioxidant protective effects on RPE cells and that blocking the alternative complement pathway activation, without restoring the activity of CFH might not be sufficient to exert full preventive and therapeutic effects in AMD.

## Materials and Methods

### Culture and treatment of ARPE-19 cells

The human retinal pigment epithelial cells ARPE-19, a no transformed human RPE line that displays many differentiated properties typical of RPE *in vivo*, were established and characterized previously^44^. ARPE-19 were grown in 6 flat bottom cell culture dishes to a confluency in a standard incubator (37 °C, 5% CO_2_) in DMEM: F12 (Invitrogen, France), supplemented with 10% calf serum, 2 mM glutamine, and 15 mM Hepes (complete culture medium). Confluent cells were cultured with medium containing 1% fetal calf serum for 2 weeks and then exposed to 30 μM 4-HNE (Merck, France) for 1, 6 or 24 h. Time zero of the kinetics corresponds to the moment of the stimulation with 4-HNE. To study the influence of CFH, cells were co-exposed to 300 nM of recCFH or one of its fragments (recCFH CCP1-7; CCP1-18; CCP8-20; CCP7-20 and rec CFH_Y402H_) produced by the Laboratoire Français du Fractionnement (LFB). Control cell cultures consist of ARPE-19 cultured in complete culture medium without treatment. Cells were washed twice with PBS 1X, detached from the flask by treatment with trypsin (Invitrogen, France), washed with complete cell culture medium and then harvested as pellet for transcriptomic or proteomic analysis.

### Culture and treatment of hiPSC-derived RPE cells

General protocol modified from Singh, R*. et al*. was used to expanse and differentiate in 60 days human induced pluripotent stem cells (hiPSC) into hiPSC-derived RPE cells (iRPE) and was recently submitted (Udry *et al*.) In brief, starting form hiPSC obtained from a healthy donor, 250 to 500 embryonic body-like aggregates were plated and cultured in P60 (60 mm) cell culture dishes coated with a matrigel matrix (Corning). Following 30 days of differentiation, pigmented foci were micro dissected, collected, seeded in matrigel-coated P60 cell culture dishes and grown for an additional 30 days. Mature pigmented RPE patches were micro dissected, purified by removal of non-RPE like cellular structures, dissociated with Trypsin-EDTA and reseeded in 24-well matrigel-coated plates for further expansion and maturation until passage 3 (*P3*). From cells at passage 1 (*P1*) to cells at *P3*, additional 2 to 3 months of cell culture were required. iRPE cells were incubated in a serum- and antibiotic-free retinal differentiation medium containing DMEM (high glucose, GlutaMAX Supplement, HEPES, ThermoFisher, France), Ham’s F-12 Nutrient Mix (ThermoFisher) (3:1 ratio) and 2% B-27 supplement minus vitamin A (ThermoFisher, France). Characterization of iRPE cells and experimentations took place at *P3* on day 42. iRPE cells cultured on transwell plates were characterized and compared to human fetal RPE and postmortem human RPE controls (Udry *et al*., submitted). The expression of specific RPE markers was assessed by RT-PCR, RT-qPCR and immunofluorescence. iRPE cells grew in monolayers of polygonal pigmented cells, demonstrated specific RPE markers expression and generated high TER levels (300 Ω • cm^2^). For experimentations, iRPE cells were seeded and grown at *P3* in 6-well (cell viability assay, semi quantitative Western blot analysis and RT-qPCR) or 24-well (immunocytochemistry) cell culture plates for 42 days. One week prior to experimentations, 1% fetal bovine serum was added to cell culture medium. At day 42, iRPE cells were treated for 6 h with 30 μM 4-hydroxy-2-nonenal (4-HNE) (Merck, France) ± 300 nM recCFH (Laboratoire français du fractionnement et des biotechnologies LFB, France). Untreated cells served as control.

### Cell viability assays

Cell viability was assessed by counting trypan blue-excluding cells after adding 0.5% trypan blue and by monitoring LDH (lactate dehydrogenase) release into the culture, with a cytotoxicity detection kit (Roche Diagnostics, Meylan, France) according to manufacturer’s recommendations. A micro plate reader calibrated with 600 and 490 nm directly measured the absorbance.

#### Measurement of mitochondrial redox potential

Mitochondrial redox potential was assessed spectrophotometrically with an MTT assay (Sigma‐Aldrich, France). Cells were seeded at 20 000 cells per well in a 12‐well plate. At 30 days after the final culture medium change, cells were stimulated with 4-HNE or both 4-HNE and recCFH for 6 hours. After cell stimulation, cells were washed once with PBS pre‐warmed to 37 °C and incubated at 37 °C in 5% CO_2_ in a solution of MTT (1 mg mL^−1^ in PBS) pre‐warmed to 37 °C. After 1 h, isopropanol (final concentration 50%) was directly added to the MTT solution, and the 12‐well plates were slowly rotated for 10 min at room temperature. The absorbance was directly measured at 570 nm in a microplate reader.

### Transmission electron microscopy

ARPE-19 cells were fixed in 2.5% glutaraldehyde cacodylate buffer (0.1 M, pH 7.4) and then fixed in 1% osmium tetroxyde in cacodylate buffer (0.2 M, pH 7.4) and progressively dehydrated in graduated ethanol solution and finally in propylene oxide. Cells were contrasted by uranyl acetate and analyzed with a transmission electron microscope (Philips CM10).

### Tunnel experiments

Apoptotic cells were visualized by the terminal deoxynucleotidyl transferase-mediated dUTP end-labeling (TUNEL) technique using the Dead End Colorimetric TUNEL system (Promega, France) after 6 hours of 4HNE and/or recCFH co-treatments.

### Immunohistochemistry

For immunohistochemical studies, ARPE-19 or iRPE cells were fixed for 15 min at 4 °C with paraformaldehyde 4% diluted in PBS solution, incubated in PBS/BSA 0.1%, permeabilized in 0.1% Triton X-100 for 20 min, saturated with normal goat serum (Cliniscience, France) 10%/PBS for 30 min at 4 °C and then stained one night at room temperature in selective primary antibodies diluted in 0.2% Triton X-100 in PBS: anti-C5b9 (1:500, rabbit, Abcam, France), anti- C3 (1:300, rabbit, Invitrogen, France), anti-C3 fragments (1:100, mouse, Hycult, France), anti-ZO-1 (1:300, rabbit, Invitrogen, France), Phalloidin (1:700, Invitrogen, France), anti-Caspase3 (1:700, rabbit, Santa-Cruz, France), anti-Caspase3 active (1 :700, rabbit, BD Biosciences, France) and anti-Caspase9 (1:700, rabbit, Cell signaling, France) and anti-CFH (1 :700, mouse, R&D system, France). After three washes in PBS/triton 0.1%, ARPE-19 cells were incubated in a solution of 1:200 of secondary antibody conjugated to Alexa (red 594 nm or green 488 nm; Molecular Probes, Interchim, France) and corresponding to the primary antibody for respectively 60 min at room temperature. The slides were then washed, stained for 5–10 min with DAPI, and washed again in PBS. Slides in the plastic labtek were then mounted with Dako solution (Dako, France) and then examined with a Zeiss confocal Imaging system (LSM710, Zeiss). As a control, the primary antibody was omitted: no staining was observed in any control.

Length of ZO-1 labelled membrane fragments was measured with an ImageJ customized macro, that automatized the following steps. The images stacks of ZO-1 labelling were projected using maximum intensity projection. Linear structures were enhanced by computing the smallest eigen values of the hessian tensor thanks to the ImageJ plugin “FeatureJ” (Erik Meijering, Erasmus University Medical Center, Rotterdam, Netherlands. Plugin available from https://imagescience.org/meijering/software/featurej. Images obtained were then binarized and skeletonized. Junction points of the image skeleton, where three or more segments are branched, were then detected by a binary hit or miss operation using the ImageJ plug-in “Morphology” (G. Landini 2008). The junction points were subtracted from the image skeleton and the remaining skeleton branches were counted and measured using the Analyze Particles function of ImageJ. Those branches were considered as representative of the continuous segments of ZO-1 labeling along the cell membranes. From the measurements obtained, histograms of the segments length where build, and the amount of large segments (x µm < length < y µm) was compared between the different cell culture conditions.

### Western blot

Total protein was extracted from ARPE-19 cells. The cells were homogenized and solubilized in ice-cold PBS containing protease inhibitors. Briefly, electrophoresis was performed by SDS-PAGE 4–12% Tris-gel and the separated proteins were transferred to nitrocellulose membrane (Immobilon; Millipore, France). The blots were blocked with 5% non fat dry milk. Mouse monoclonal anti-β-actin (1:3000, mouse, Abcam, France) or RIP3(1 :2000, rabbit, ThermoFisher, France) were used as primary antibodies overnight at 4 °C, and then the blots were washed with TBS 1X/milk 1% and incubated separately with the corresponding second antibody coupled to horseradish peroxidase (1:3000, Abcam, France). Blots were developed using the enhanced chemiluminescence Western blotting detection system “ECL-Plus” (Amersham Pharmacia Biotech, Arlington Heights, IL, France) according to manufacturer’s recommendations. Quantification of RIP3 and β-actin were accomplished by analyzing the intensity of the bands using ImageJ program (National Institute of Health, Bethesda, MD).

### Quantitative real-time polymerase chain reaction (RT-qPCR)

Cellular material from at least three 6-well plate dishes was pooled for each condition. Total RNA from ARPE-19 was isolated with TRIZOL reagent (Invitrogen, France) according to the manufacturer’s instructions, and Superscript II Reverse Transcriptase (Invitrogen, France) was used to reverse transcribe 1 μg of mRNA. Amplification reaction assays contained 1 × SYBR Green PCR Mastermix (Applied Biosystems, France). All real-time PCR oligonucleotide primers were previously experimentally validated by QPCR and BLAST. Primers were designed such that amplicon sizes ranged from 50 to 250 bps (Table 1). A hot start at 95 °C for 5 min was followed by 40 cycles at 95 °C for 15 s and 60 °C for 1 min with the 7300 SDS thermal cycler (Applied Biosystems, France). Controls with no reverse transcriptase were run for each assay to confirm the lack of genomic DNA contamination. Control RT-qPCR reactions were performed without cDNA templates. *Actin* was used as a suitable reference gene. The standard curve method (Prism 7700 Sequence Detection System; ABI User Bulletin number 2) was used for relative quantification of gene expression. At least three experiments were performed for each gene and sample. For all experiments, each individual sample was run in triplicate wells and the Ct of each well was recorded at the end of the reaction. The average and standard deviation of the three Cts was calculated. Gene expression levels were normalized to actin for each cellular material sample and calculated relative to no treated culture (control) with the following equation: relative expression = 2^−(sampleΔCt − controlΔCt)^ where ΔCt = mean Ct(target) − mean Ct(actin).

**Table 1:** List of forward and reverse primers for qPCR experiments.

Gene	Forward 5′ ➝ 3′	Reverse 5′ ➝ 3′
Kir4.1	CAAGGACCTGTGGACAACCT	GGGATTCAAGGGAGAAGAGG
Kir7.1	CCCACCTGAAAACCACACTATCTG	GCATGAGGCCTAGGAGCATTTG
Aqp1	TGGACACCTCCTGGCTATTG	GGGCCAGGATGAAGTCGTAG
IL6	GATGGATGCTTCCAATCTGGAT	AGTTCTCCATAGAGAACAACATA
IL8	CGATGTCAGTGCATAAAGACA	TGAATTCTCAGCCCTCTTCAAAAA
IL1beta	CATCAGCACCTCTCAAGCAG	GAGTCCACATTCAGCACAGG
Caspase8	CTGCTGGGGATGGCCACTGTG	TCGCCTCGAGGACATCGCTCTC
Catalase	TAAGACTGACCAGGGCA	CAAACCTTGGTGAGATCGAA
Gpx	CCTCAAGTACGTCCGACCTG	CAATGTCGTTGCGGCACACC
Inos	GTTCTCAAGGCACAGGTCTC	GCAGGTCACTTATGTCACTTATC
Actin	AGGAGAAGCTTGCTACGTC	AGGGGCCGGACTCGTCATAC

### Statistical analyses

Results are presented as the mean ± SEM. Statistical analyses were performed using GraphPAD Prism 5 software. For data related to qPCR and western blot, comparison between two groups was performed using Mann–Whitney test.

## Supplementary information

CFH fragments have no toxic effect on ARPE-19 cells.

CFH preserves ARPE-19 actin structure from oxidative stress.
