# Supplementary figures and images for: CFH exerts anti-oxidant effects on retinal pigment epithelial cells independently from protecting against membrane attack complex
Source: Sci Rep. 2019 Sep 25;9:13873. doi: 10.1038/s41598-019-50420-9 (PMC6761137; doi:10.1038/s41598-019-50420-9)

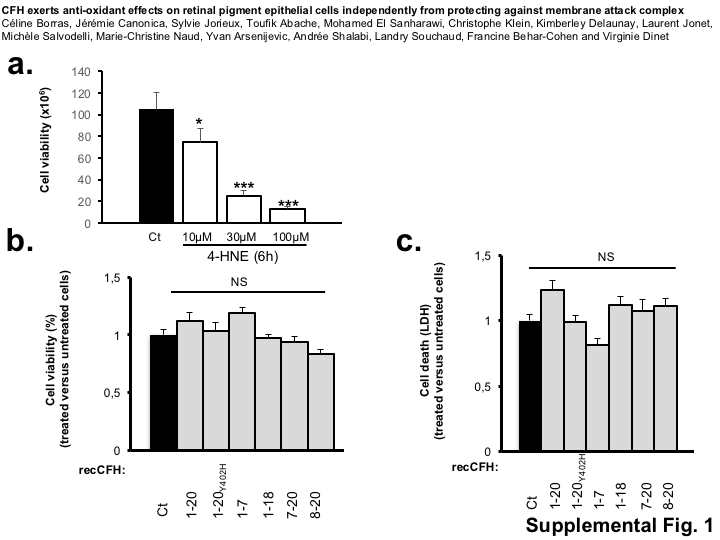

Supplement: Supplementary file 1 — CFH fragments have no toxic effect on ARPE-19 cells. [file 41598_2019_50420_MOESM1_ESM.tif]

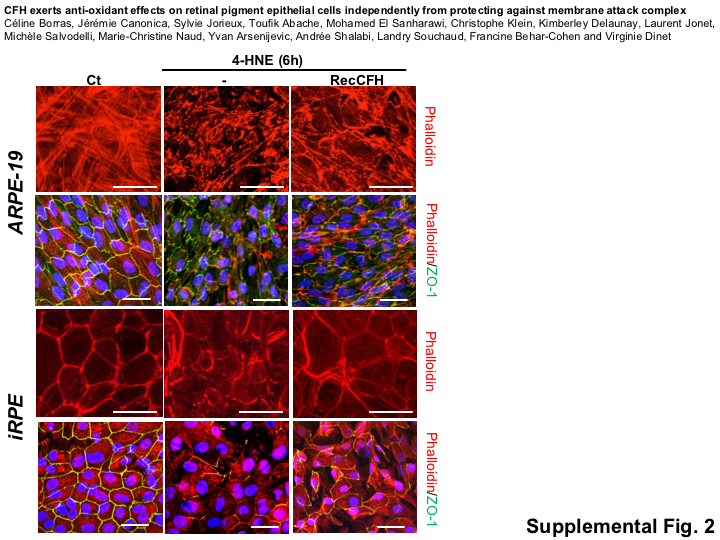

Supplement: Supplementary file 2 — CFH preserves ARPE-19 actin structure from oxidative stress. [file 41598_2019_50420_MOESM2_ESM.tif]
